# Self-reported acceptable pain intensity is associated with psychosocial burden beyond pain severity in chronic pain patients: a cross-sectional study

**DOI:** 10.1186/s40359-026-05731-8

**Published:** 2026-10-13

**Authors:** Yannick Bruns, Jorge Manuel, Alexander Neumann, Mirjam Eberhardt, Matthias Karst

**Affiliations:** 1 Department of Anaesthesiology and Intensive Care Medicine, Pain Clinic, Hannover Medical School, Carl-Neuberg-Str. 1, 30625 Hannover, Germany; 2 Institute for Neuroradiology, Hannover Medical School, Hannover, Germany; 3 Institute of Aerospace Medicine, German Aerospace Center (DLR), Cologne, Germany; 4 Institute for Neuropathology, University Medical Center Göttingen, Göttingen, Germany

**Keywords:** Acceptable pain, Chronic pain, Psychosocial burden, Somatization, Sleep impairment, Pain assessment

## Abstract

**Background:**

Self-reported acceptable pain intensity is collected as part of intake procedures in some pain clinic settings, yet has rarely been examined in relation to psychosocial outcomes. This study investigated whether acceptable pain intensity is associated with psychosocial symptom burden (somatization, anxiety, sleep impairment, perceived stress, depression, and pain-related disability) in chronic pain patients after adjusting for pain severity and trauma history.

**Methods:**

Secondary analysis of 822 chronic pain patients from a German university pain clinic (previously published cohort). Acceptable pain (visual analogue scale [VAS], 0-100) was examined in relation to somatization (PHQ-15), anxiety (GAD-7), sleep impairment (VAS), perceived stress (PHQ-Stress), depression (PHQ-9), and disability (Pain Disability Index) in multivariable regression models adjusted for three pain intensity measures, trauma category, age, and sex. Acceptable pain was scaled per 10-point VAS increment. Bonferroni correction was applied (α = 0.008). Post-hoc trauma × acceptable pain interactions were tested.

**Results:**

Higher acceptable pain was associated with greater somatization (B = 0.37, 95% CI 0.19–0.55, *p* < .001), anxiety (B = 0.30, 0.11–0.48, *p* = .002), sleep impairment (B = 1.75, 0.67–2.84, *p* = .002), and perceived stress (B = 0.23, 0.09–0.37, *p* = .002), but not with depression (*p* = .054) or disability (*p* = .82). Findings were consistent across three prespecified sensitivity analyses. An exploratory post-hoc interaction test suggested that the acceptable pain-depression association was more pronounced in higher trauma categories (Bonferroni-significant trauma × acceptable pain interaction, *p* < .001).

**Conclusions:**

Higher self-reported acceptable pain intensity is associated with elevated psychosocial symptom burden beyond pain severity, with significant adjusted associations for somatization, anxiety, sleep impairment, and perceived stress, but not for depression or disability. Although the incremental contribution was small, the direction of the association was consistent across the four significant outcomes. Higher acceptable pain should therefore not be interpreted as evidence of better coping. Instead, it may reflect heterogeneous processes such as low outcome expectations, resigned tolerability, or psychosocial vulnerability, particularly among patients in higher trauma categories; shared method variance cannot be excluded. Prospective validation is needed.

**Supplementary Information:**

The online version contains supplementary material available at https://doi.org/10.1186/s40359-026-05731-8.

## Background

Pain intensity is the most commonly assessed dimension in chronic pain, yet psychosocial factors, including depression, anxiety, catastrophizing, and sleep disturbance, account for substantial variance in pain-related outcomes [1–4]. Pain catastrophizing has emerged as one of the strongest psychological predictors of pain-related distress [5], and outcome expectations similarly shape treatment outcomes [6, 7]. The identification of readily available clinical markers that help detect patients with elevated psychosocial burden remains an important goal for pain medicine.

Self-reported acceptable pain intensity (i.e., the pain level a patient would consider tolerable) is used in some pain clinic settings as part of intake procedures using a single visual analogue scale (VAS) item. Only a small number of studies have examined patient-defined acceptable treatment outcomes in chronic pain [8], and a position paper by Forget et al. [9] concluded that the meaning of acceptable pain likely differs between individuals and depends on contextual factors that current quantitative measures cannot sufficiently capture. Despite being collected alongside standard assessment batteries, this variable has rarely been examined in relation to patient outcomes. It exists in conceptual proximity to, but is distinct from, several established constructs in pain research.

Pain acceptance, as measured by the Chronic Pain Acceptance Questionnaire (CPAQ), captures willingness to experience pain while engaging in valued activities and is associated with lower distress [10, 11]. The CPAQ was not available in the present dataset and is included here as a conceptual reference point only. The Patient Acceptable Symptom State (PASS) defines a threshold at which patients consider their condition satisfactory, used primarily as a treatment-response criterion [12, 13]. Pain tolerance, as assessed by the Heidelberg Pain Chronification Screening (HKF-R 10), reflects expectations of how much pain would be bearable and predicts chronification in low back pain [14, 15]. Self-reported acceptable pain intensity as a single VAS item captures none of these constructs specifically; rather, it reflects the patient’s subjective threshold of tolerability.

Intuitively, one might expect higher acceptable pain to be a favorable indicator, reflecting greater resilience or better coping. However, an alternative interpretation is equally plausible: patients chronically exposed to high pain and psychosocial burden may recalibrate their internal standards upward, coming to accept higher pain levels not because they cope well but because they have adapted to suffering. Such internal standard recalibration has been described in the response shift literature in quality-of-life research [16, 17], although its application to cross-sectional pain data has not been empirically tested. Alternatively, patients who report high acceptable pain may express low expectations of improvement, a pattern associated with poorer treatment outcomes [6, 7].

We previously characterized a cohort of 914 chronic pain patients with respect to trauma category and indicators of central sensitization [18]. This dataset includes acceptable pain alongside comprehensive psychosocial assessment instruments. The aim of the present study was to examine whether self-reported acceptable pain intensity is associated with psychosocial symptom burden after adjusting for actual pain intensity and trauma history. The term psychosocial symptom burden is used here as an overarching description of six separately assessed outcome domains (somatization, anxiety, sleep impairment, perceived stress, depression, and pain-related disability) and does not imply a composite score or a common latent construct. Given the exploratory nature of this analysis, no directional hypothesis was specified a priori.

## Methods

### Study design and setting

This study is a secondary analysis of a previously published cross-sectional dataset [18]. Data were collected from February 2019 to July 2020 at the outpatient pain clinic of Hannover Medical School, Germany. The study was approved by the Hannover Medical School ethics committee (No. 9681_BO_K_2021, approved 18 March 2021) and registered at ClinicalTrials.gov (NCT05190367). Reporting follows the STROBE checklist for cross-sectional studies [19].

### Participants

The original cohort comprised 914 consecutive chronic pain patients [18]. For the present analysis, we included all patients with complete data on all study variables and a classifiable trauma history, yielding an analytic sample of *n* = 822. Exclusions were: unclassifiable trauma category (*n* = 91, per the original study protocol) and missing data on any study variable (*n* = 1). Chronic pain was defined as pain persisting for more than three months; there were no restrictions on pain diagnosis. All data were baseline data, i.e., they were collected at the time of the patient’s first visit to the outpatient pain clinic.

### Measures

All data were collected via the SymptomMapper application, a validated digital assessment tool for pain clinic settings [20]. The application requires sequential item completion before submission, which accounts for the near-complete data availability in the present sample (missing data on any variable: *n* = 1).

#### Predictor variable

Acceptable pain intensity was assessed using a single VAS item (0-100; 0 = no pain, 100 = maximum imaginable pain): “Welche Schmerzintensität wäre für Sie akzeptabel?” (“Which pain intensity would be acceptable for you?”). For interpretability of regression coefficients, this variable was scaled per 10-point VAS increment.

####  Adjustment variables

Mean pain intensity over the past four weeks (VAS, 0-100; 0 = no pain, 100 = maximum imaginable pain), maximal pain intensity over the past four weeks (VAS, 0-100), current pain intensity (VAS, 0-100), trauma category (four-level ordinal: 0 = no trauma, 1 = accidental trauma, 2 = interpersonal trauma, 3 = trauma with PTSD; classified per [18]), age (years), and sex (female/male). The ordinal coding reflects an assumed monotonic ordering of trauma category and is consistent with the original study protocol [18]; a sensitivity analysis using dummy-coded trauma variables was prespecified and is reported below.

####  Outcome variables

Primary outcome: somatization (Patient Health Questionnaire somatic symptom severity scale, PHQ-15; range 0–30) [21]. Secondary outcomes: anxiety (Generalized Anxiety Disorder scale, GAD-7; range 0–21) [22], sleep impairment (VAS, 0-100), perceived stress (PHQ-Stress module; range 0–20), depression (Patient Health Questionnaire depression scale, PHQ-9; range 0–27) [23], and pain-related disability (Pain Disability Index, PDI; range 0–70) [24]. All standardized questionnaires have established validity and reliability documented in the cited validation studies and have been used extensively in chronic pain populations. Somatization was designated a priori as the primary outcome because somatic symptom burden is the psychosocial dimension most conceptually proximal to a self-reported judgement about the tolerability of bodily sensation, and because it is a core clinical concern in chronic pain populations; this designation was made on conceptual grounds, independently of the observed associations.

### Statistical analysis

Descriptive statistics (mean, standard deviation, median) were computed for all study variables. Bivariate Pearson correlations were calculated between acceptable pain and all outcomes.

Multivariable linear regression models were computed for each outcome, with age, sex, mean pain intensity, maximal pain intensity, current pain intensity, trauma category, and acceptable pain intensity (per 10 VAS points) entered simultaneously. The incremental variance (ΔR²) attributable to acceptable pain was determined by comparing the full model to a base model without acceptable pain (F-change test). Partial correlations between acceptable pain and each outcome, adjusted for all covariates, were computed for graphical display. Variance inflation factors (VIF) were calculated to assess multicollinearity.

To account for multiple comparisons across six outcomes, Bonferroni correction was applied (adjusted α = 0.008). Benjamini-Hochberg false discovery rate (FDR) correction was additionally reported in the supplement.

Three prespecified sensitivity analyses were conducted: (a) replacement of three pain intensity measures with mean pain only, (b) exclusion of extreme acceptable pain values (VAS = 0 and VAS > 80; *n* = 745 included), and (c) replacement of ordinal trauma category with dummy-coded variables (reference: no trauma). All analyses were performed using Python 3.11.

Two additional post-hoc analyses, clearly labeled as exploratory, were conducted to clarify aspects of the primary findings. First, to test whether the association between acceptable pain and psychosocial outcomes was moderated by trauma category, multiplicative interaction terms (trauma category × acceptable pain) were added to each of the six primary models. Bonferroni correction across the six interaction tests was applied at α = 0.008. Second, to characterize the asymmetric distribution of acceptable pain values, three subgroups were defined post-hoc, for descriptive purposes, based on the distribution of acceptable pain values reported in Sample Characteristics (Acc = 0, *n* = 58; Acc 1–49, *n* = 698; Acc ≥ 50, *n* = 66) and compared on demographic, pain, trauma, and psychosocial variables using Welch’s t-tests against the Acc 1–49 reference group.

In summary, the primary outcome, the covariate set, and the three sensitivity analyses described above were specified before the results were examined. The trauma category × acceptable pain interaction and the acceptable pain subgroup comparisons were exploratory and post hoc, undertaken after inspection of the primary results to help interpret them; no directional hypothesis for the primary association was specified in advance.

The sample size was determined by the available data from the parent study and was not based on an a priori power calculation for the present analysis. Post-hoc power analysis indicated that with *n* = 822 and eight predictors at α = 0.008, the study had 93% power for the largest observed effect (somatization, f² = 0.021) and approximately 69–70% power for the smaller significant effects (anxiety, sleep, stress; f² ≈ 0.012).

## Results

### Sample characteristics

The analytic sample comprised 822 patients (68.1% female; mean age 53.7 ± 16.2 years). Mean pain intensity was 59.2 ± 22.8, and mean acceptable pain was 25.5 ± 19.1 (VAS). Acceptable pain was positively skewed (skewness = 1.21): 7.1% of patients reported an acceptable pain level of 0 (“no pain acceptable”), while 8.0% reported values ≥ 50. Regarding trauma, 28.1% had no trauma history, 25.5% accidental, 34.3% interpersonal, and 12.0% PTSD-level trauma. Full sample characteristics are shown in Table 1.

**Table 1:** Sample characteristics and bivariate correlations with acceptable pain intensity (*n* = 822)

Variable	Mean	SD	Median	*r*
Age (years)	53.7	16.2	55.0	
Female sex, n (%)	560 (68.1)	—	—	
Mean pain (VAS)	59.2	22.8	60.0	0.35***
Maximal pain (VAS)	76.1	21.4	81.0	0.29***
Current pain (VAS)	52.3	28.0	55.0	0.38***
Acceptable pain (VAS)	25.5	19.1	23.0	—
Somatization (PHQ-15)	12.5	5.4	12.0	0.29***
Anxiety (GAD-7)	7.9	5.2	7.0	0.24***
Sleep impairment (VAS)	52.9	32.6	54.0	0.29***
Perceived stress (PHQ-Stress)	6.7	4.1	6.0	0.21***
Depression (PHQ-9)	11.2	5.7	11.0	0.24***
Disability (PDI)	36.0	15.4	36.0	0.22***
Trauma category, n (%)				
None	231 (28.1)	—	—	
Accidental	210 (25.5)	—	—	
Interpersonal	282 (34.3)	—	—	
PTSD	99 (12.0)	—	—	

Note. r = bivariate Pearson correlation with acceptable pain intensity. VAS = visual analogue scale (0-100). ***p < .001

Acceptable pain was moderately positively correlated with mean pain intensity (*r* = .35), maximal pain (*r* = .29), and current pain (*r* = .38). Bivariate correlations with psychosocial outcomes ranged from *r* = .21 (PHQ-Stress) to *r* = .29 (PHQ-15); the correlation with disability (PDI) was *r* = .22 (Table 1).

### Multivariable regression analyses

Table 2 presents the regression models for all six outcomes. Across all models, trauma category showed consistently significant associations with the psychosocial outcomes (unstandardized B ranging from 1.29 to 3.43 across outcomes, reflecting different outcome scales), alongside mean pain intensity and acceptable pain.

**Table 2:** Multivariable linear regression: predictors of psychosocial outcomes and disability (*n* = 822). Models adjusted for mean, maximal, and current pain intensity, trauma category, age, and sex

Predictor	PHQ-15	GAD-7	Sleep VAS	PHQ-Stress	PHQ-9	PDI
	B [95% CI]	B [95% CI]	B [95% CI]	B [95% CI]	B [95% CI]	B [95% CI]
Age	0.01 [− 0.01, 0.03]	−0.03* [− 0.05, − 0.01]	−0.14* [− 0.27, − 0.02]	−0.02* [− 0.04, − 0.003]	−0.03* [− 0.05, − 0.01]	−0.01 [− 0.07, 0.05]
Female sex	1.03** [0.36, 1.71]	0.69† [− 0.00, 1.38]	−1.66 [− 5.74, 2.42]	0.14 [− 0.41, 0.69]	0.26 [− 0.45, 0.98]	−0.56 [− 2.44, 1.32]
Mean pain	0.03* [0.004, 0.05]	0.04*** [0.02, 0.07]	0.44*** [0.29, 0.59]	0.02 [− 0.004, 0.04]	0.06*** [0.04, 0.09]	0.25*** [0.18, 0.32]
Maximal pain	0.03* [0.01, 0.05]	−0.01 [− 0.03, 0.01]	0.09 [− 0.04, 0.23]	−0.00 [− 0.02, 0.02]	0.01 [− 0.01, 0.03]	0.07* [0.01, 0.13]
Current pain	0.03** [0.01, 0.04]	0.02* [0.003, 0.04]	0.16** [0.05, 0.26]	0.01 [− 0.004, 0.03]	0.03** [0.01, 0.05]	0.06* [0.01, 0.11]
Trauma category	1.55*** [1.23, 1.87]	1.29*** [0.96, 1.62]	3.43*** [1.50, 5.37]	1.39*** [1.14, 1.65]	1.37*** [1.03, 1.71]	2.35*** [1.46, 3.24]
Acceptable pain	0.37*** [0.19, 0.55]	0.30** [0.11, 0.48]	1.75** [0.67, 2.84]	0.23** [0.09, 0.37]	0.19† [− 0.00, 0.38]	0.06 [− 0.44, 0.55]
R²	0.283	0.193	0.283	0.202	0.267	0.326
ΔR² (acc. pain)	0.014	0.009	0.008	0.010	0.003	< 0.001

Note*.* B = unstandardized regression coefficient [95% confidence interval]. Acceptable pain (acc. pain) scaled per 10 VAS points; pain measures per 1 VAS point; trauma per ordinal category. Significance symbols denote uncorrected p-values. Bonferroni correction (α = 0.008) was applied to inference for the primary predictor (acceptable pain) across the six outcome models. ***p < .001; **p < .01; *p < .05; †*p* < .10

After adjustment for age, sex, three pain intensity measures (mean, maximal, current), and trauma category, acceptable pain intensity (per 10 VAS points) was significantly associated with somatization (B = 0.37, 95% CI 0.19–0.55, *p* < .001), anxiety (B = 0.30, 0.11–0.48, *p* = .002), sleep impairment (B = 1.75, 0.67–2.84, *p* = .002), and perceived stress (B = 0.23, 0.09–0.37, *p* = .002). The association with depression showed no significance (B = 0.19, − 0.00-0.38, *p* = .054), and there was no association with disability (B = 0.06, − 0.44–0.55, *p* = .82).

Incremental variance explained by acceptable pain (ΔR²) was small, ranging from 0.8% to 1.4% for the four significant outcomes, and was negligible for depression (0.3%) and disability (< 0.1%). These four associations also showed significance after Benjamini-Hochberg FDR correction (Additional file 1: Table S1). All variance inflation factors were ≤ 3.3 (acceptable pain: 1.19), indicating that multicollinearity with the pain intensity measures was not a concern.

### Partial correlations

**Fig. 1:**
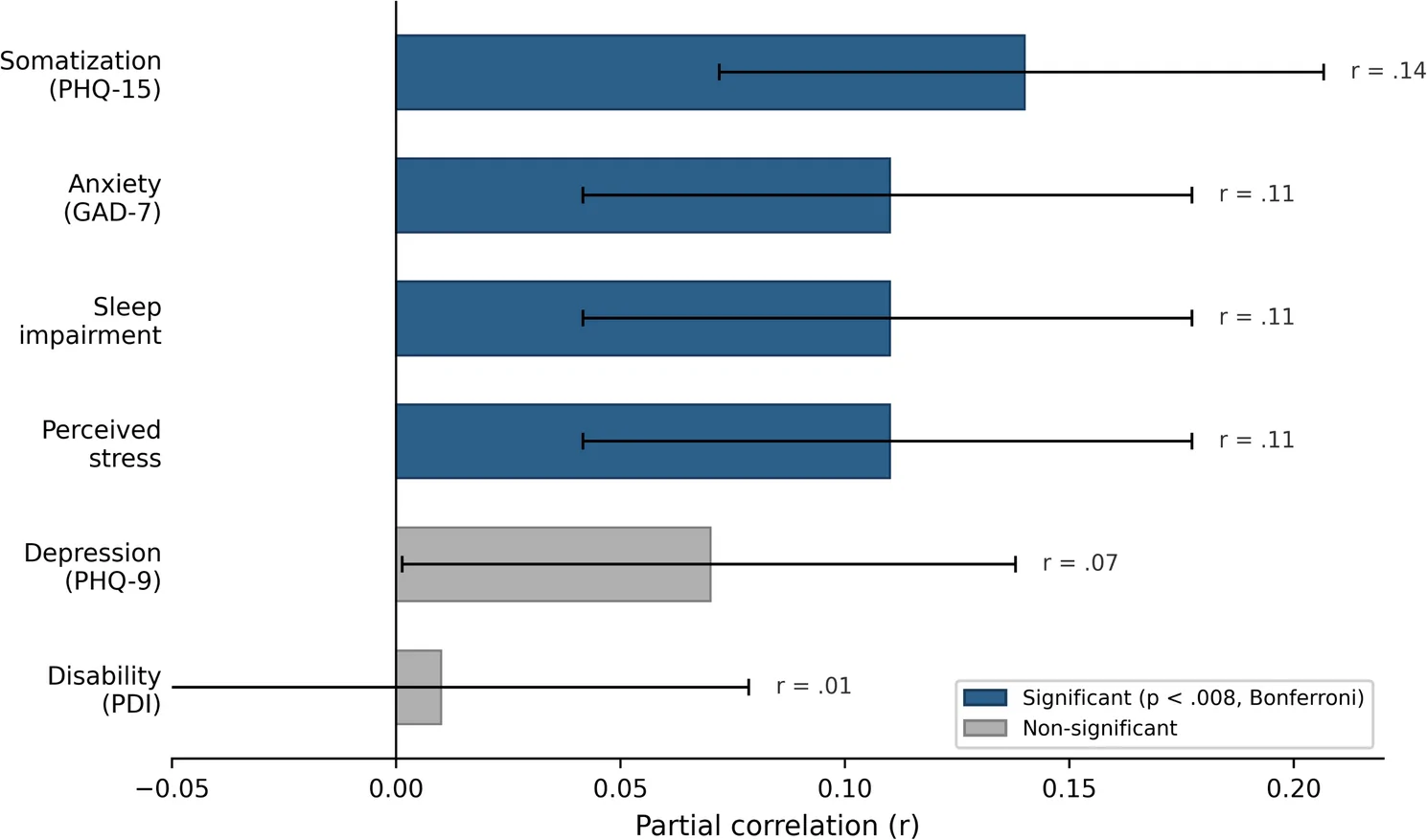
Partial correlations between self-reported acceptable pain intensity and six psychosocial outcomes, adjusted for mean, maximal, and current pain intensity, trauma category, age, and sex (*n* = 822). Acceptable pain was scaled per 10 VAS points. Error bars indicate 95% confidence intervals (Fisher z-transformation). Blue bars indicate associations significant after Bonferroni correction (α = 0.008); grey bars indicate non-significant associations

Figure 1 displays partial positive correlations between acceptable pain and each outcome after adjustment for all covariates. Partial correlations were *r* = .14 for somatization, *r* = .11 for anxiety, sleep impairment, and stress, *r* = .07 for depression, and *r* = .01 for disability. The dissociation between psychosocial outcomes (significant) and disability (non-significant) was the most prominent pattern.

### Sensitivity analyses

Results were consistent across all sensitivity analyses (Additional file 1: Table S2). When only mean pain intensity (rather than three pain measures) was used as covariate, acceptable pain effects were slightly larger but followed the same pattern: somatization (B = 0.43, *p* < .001), anxiety (B = 0.33, *p* < .001), sleep impairment (B = 2.11, *p* < .001), and stress (B = 0.26, *p* < .001). When trauma was dummy-coded (vs. ordinal), all four significant associations remained significant. When extreme acceptable pain values were excluded (*n* = 745), somatization (*p* < .001) and sleep impairment (*p* < .001) remained highly significant; anxiety and stress remained significant at α = 0.05 (*p* = .024 and *p* = .007, respectively). Across all sensitivity specifications, disability (PDI) remained non-significant.

### Post-hoc analyses

#### Trauma × acceptable pain interaction

A multiplicative interaction term (trauma category × acceptable pain per 10 VAS) was added to each of the six primary models. Of the six interaction tests, the interaction reached significance only for depression (PHQ-9: B = 0.34, 95% CI [0.17, 0.50], *p* < .001, ΔR² = 0.014), surviving Bonferroni correction across the six interaction tests (α = 0.008). Stratified estimates clarified the pattern: in patients without trauma, the acceptable pain coefficient for PHQ-9 was B = − 0.05 (95% CI [− 0.42, 0.32]); accidental trauma B = − 0.03 [− 0.39, 0.33]; interpersonal trauma B = + 0.35 [0.00, 0.70]; PTSD-level trauma B = + 0.67 [0.19, 1.15]. A parallel pattern was observed for somatization, with the largest stratum-specific coefficients in the no-trauma and PTSD groups (PHQ-15: B = + 0.40 and B = + 0.72, respectively), although the omnibus interaction did not survive correction (*p* = .107). Interaction terms for the remaining four outcomes were not significant after correction (Additional file 1: Table S3).

#### Acceptable pain subgroups

Three subgroups defined by acceptable pain value were compared (Acc = 0, *n* = 58; Acc 1–49, *n* = 698; Acc ≥ 50, *n* = 66). Relative to the Acc 1–49 reference, the Acc = 0 subgroup was younger (mean 46.1 vs. 54.4 years, *p* = .002), reported a lower proportion of PTSD-level trauma (8.6% vs. 11.3%), and showed lower PHQ-15 (10.1 vs. 12.4, *p* = .002), GAD-7 (6.0 vs. 7.8, *p* = .007), and PHQ-Stress (5.1 vs. 6.7, *p* = .002) scores. The Acc ≥ 50 subgroup showed the opposite pattern, with the highest proportion of PTSD-level trauma (22.7%), the highest mean pain (77.2 vs. 57.8), and elevated scores across all psychosocial outcomes (PHQ-15: 15.8; PHQ-9: 14.6; Additional file 1: Table S4). These subgroup differences persisted after adjustment for all three pain intensity measures for all psychosocial outcomes except disability, indicating that they are not attributable to higher pain intensity alone. The psychosocial burden associated with acceptable pain was thus concentrated at the upper end of the distribution rather than distributed symmetrically across it.

## Discussion

### Summary of findings

Self-reported acceptable pain intensity was associated with psychosocial symptom burden, specifically somatization, anxiety, sleep impairment, and perceived stress, but not with depression or pain-related disability, after adjustment for three pain intensity measures and trauma history. The direction of this association was contrary to what a favorable interpretation of acceptable pain would predict: patients who reported higher acceptable pain showed more, not less, psychosocial burden.

### Interpretation

Several non-exclusive mechanisms may explain the observed association, and the cross-sectional design does not allow definitive adjudication among them.

One possible framework for this expectation- and control-related interpretation is the Cognitive Activation Theory of Stress, which conceptualizes stress responses in relation to outcome expectancies, perceived coping capacity, and perceived control [25]. Within this framework, higher acceptable pain may indicate not adaptive acceptance, but a resigned expectation that substantial improvement is unlikely. However, because the present study is cross-sectional and did not directly assess expectations or perceived control, this interpretation remains hypothetical.

First, higher acceptable pain may reflect low outcome expectations. The question can be read as an implicit expectation item: patients who report high acceptable pain may express that they do not expect substantial improvement. As low treatment expectations are associated with poorer outcomes in chronic pain [6, 7], high acceptable pain would signal demoralization rather than coping capacity.

Second, higher acceptable pain may reflect resignative recalibration of internal standards. Patients chronically exposed to both high pain and psychosocial distress may shift their reference frame upward. This interpretation draws on response shift theory [16, 17], although as noted in the introduction, response shift is inherently a longitudinal concept. Recalibration response shift has been demonstrated for pain ratings in cancer surgery populations [26], but direct evidence in non-malignant chronic pain is lacking. In the present dataset, trauma category was independently associated with higher acceptable pain after adjustment for all three pain intensity measures, age, and sex (B = 1.31, 95% CI [0.09, 2.52], *p* = .036, ΔR² = 0.5%), consistent with trauma-related upward recalibration of pain tolerance thresholds. A post-hoc test for moderation supports this reading: the acceptable pain - depression association (PHQ-9) was more pronounced in higher trauma categories, with stratum-specific coefficients of B ≈ 0 in the no-trauma and accidental-trauma groups rising monotonically to B = + 0.67 per 10 VAS in patients with PTSD-level trauma (Bonferroni-significant trauma × acceptable pain interaction; see Results, Post-hoc Analyses). The asymmetric concentration of psychosocial burden in the high acceptable pain subgroup, which contained a disproportionate share of PTSD-level trauma, points in the same direction. Both observations are post hoc and require replication. They are compatible with the possibility that acceptable pain ratings have different meanings across trauma categories, but they do not establish a trauma-related mechanism and cannot rule out shared reporting tendencies.

Third, a methodological alternative must be considered. Negative affectivity (NA) is known to inflate correlations between self-report measures [27]. The specificity pattern in our data (significant associations with psychosocial outcomes but not with disability) is consistent with NA-related reporting bias, as the PDI captures functional limitations that may be less susceptible to such bias. We cannot exclude that shared method variance partly accounts for the observed associations. The adjustment for three correlated pain intensity measures attenuates but does not eliminate this concern, as pain ratings themselves are subject to NA bias.

Fourth, research using the Heidelberg Pain Chronification Screening (HKF-R 10) has shown that low pain tolerance predicts chronification of acute back pain [14, 15]. Our finding may represent a complementary observation in already-chronified pain patients. However, this extrapolation must be treated cautiously, as it involves different populations, instruments, and potentially different constructs. The absence of an association with disability likely reflects that the PDI primarily captures functional limitations determined by pain intensity, which was fully adjusted for.

The four significant associations, with somatization, anxiety, sleep impairment, and perceived stress, showed a similar direction and magnitude, which is compatible with a shared underlying process rather than four independent mechanisms. One hypothesis for future research is that a single latent factor, such as general psychological distress or negative affectivity, underlies the association with all four outcomes, whereas depression and disability showed weaker or absent independent associations in the present models. Testing this would require a model examining the four correlated outcomes jointly rather than in separate regressions, which was beyond the scope of the present study and is proposed here as a direction for future work rather than an additional analysis of the current data. Somatization, the primary outcome, may be particularly closely linked to acceptable pain because both concern the interpretation of bodily sensation. The associations with anxiety and perceived stress are consistent with a general distress interpretation, the sleep-related association may additionally reflect arousal, fatigue, or sleep-related distress, but this cannot be distinguished with the present single-item sleep measure. These explanations remain hypotheses for future research and should not be read as established mechanisms.

### Relationship to established constructs

The weak effect sizes observed are consistent with the conceptual heterogeneity of the construct itself. Forget et al. [9] concluded in a position paper that the meaning of “acceptable pain” likely differs between individuals and depends on contextual factors that quantitative VAS scores cannot sufficiently capture. Such heterogeneity at the construct level would attenuate regression associations even in the presence of genuine relationships, and may partly account for the small but consistent incremental variance observed here. Consistent with this framing, responses to a single-item acceptable pain VAS may reflect treatment expectations, normative beliefs about pain endurance, resigned tolerance, or strategic communication within a clinical encounter. These distinct meanings cannot be disentangled by the present design. This interpretation also aligns with the semantic ambiguity discussed in the limitations section below.

The acceptable pain VAS item showed a positive association with psychosocial burden, in contrast to CPAQ-based pain acceptance (which was not available in this dataset but is included here as a conceptual reference), which is negatively associated with distress [10, 11]. Kranz et al. [28] further demonstrated that the two CPAQ subcomponents have differentiated affective correlates: pain willingness primarily reduces negative affect, whereas activity engagement additionally promotes positive affect. This specificity contrasts sharply with the unspecific pattern in our data, where higher acceptable pain was associated with elevated scores across the four Bonferroni-significant psychosocial outcomes, with a numerically similar but non-significant association for depression. The opposite directionality and lack of specificity suggest a fundamentally different construct. We hypothesize that acceptable pain intensity reflects a tolerance threshold rather than a coping quality, and that these may be inversely related. An alternative reading cannot be fully excluded: patients who report high acceptable pain alongside elevated distress might be those who, despite considerable burden, continue to engage with their situation rather than withdraw, which could reflect an aspect of resilience in the sense of continued functioning under adversity. This further suggests that the construct captured by a single acceptable pain VAS item is heterogeneous, and that distinguishing resignation from resilience will require concurrent measurement of coping processes in future studies. This hypothesis requires formal testing through concurrent assessment of the CPAQ and the acceptable pain VAS item. Similarly, the applicability of the PASS concept, developed primarily in acute and perioperative settings, to chronified pain populations warrants separate investigation.

Several constructs that may mediate or moderate the observed association were not assessed, including pain catastrophizing and pain self-efficacy. Their absence limits interpretive specificity and constitutes a priority for future replication.

### Clinical implications

Acceptable pain intensity is already collected in some pain settings. The present data suggest that a high value should not be interpreted uncritically as a favorable prognostic sign. Rather, in the context of a comprehensive assessment, it may prompt clinicians to consider additional psychosocial evaluation, particularly in patients who appear to function adequately despite high pain levels. However, given the small incremental variance (ΔR² = 0.8–1.4% for the four Bonferroni-significant outcomes), these observations are hypothesis-generating and do not establish acceptable pain as a screening criterion; whether this readily available item has incremental clinical value remains to be tested prospectively.

To provide descriptive context: patients reporting high acceptable pain (≥ 50; *n* = 66) had a mean PHQ-15 score of 15.8, compared with 10.1 in patients for whom no pain was acceptable (acceptable pain = 0; *n* = 58), a difference of more than five points, spanning from the moderate to the severe somatic symptom range (severity thresholds at 5, 10, 15 [21]; Additional file 1: Table S4). These subgroup figures are unadjusted and are presented for descriptive context only.

### Limitations

Several limitations must be considered. First, the cross-sectional design precludes causal inference. Second, acceptable pain was assessed with a single VAS item with no established psychometric properties. The item is semantically ambiguous: responses may reflect treatment expectations, resignation to ongoing suffering, normative beliefs about pain endurance, or functional trade-offs, and the present data cannot distinguish among these interpretations. A related limitation concerns the sleep impairment outcome, which was likewise assessed with a single visual analogue scale rather than a multidimensional, validated sleep instrument such as the Pittsburgh Sleep Quality Index [29] or the Insomnia Severity Index [30]; this does not invalidate the analysis but constrains the interpretation of the sleep-related finding. Third, although the primary outcome and the associated statistical models were specified before the results were examined, no directional hypothesis was formulated in advance for this exploratory secondary analysis, and the non-significant association with depression (*p* = .054) may reflect insufficient power (~ 24% at the corrected threshold) rather than absence of a true effect. Fourth, important potential confounders were not available, including pain diagnoses, pain duration, medication use, and psychiatric comorbidities. The absence of a measure of pain catastrophizing is particularly relevant, as catastrophizing is a strong cognitive predictor of pain-related distress [5]. Fifth, the absence of a measure of trait negative affectivity prevents quantifying shared method variance [27]. Future studies should include trait measures of NA (e.g., the Negative Affect Schedule). Sixth, the sample comprises tertiary care patients from a single German center, limiting generalizability. Seventh, the trauma × acceptable pain interaction analysis and the acceptable pain subgroup comparisons reported under Post-hoc Analyses were not prespecified; they are exploratory in nature, were used to clarify rather than establish the primary findings, and require independent replication.

### Future directions

Future studies should examine whether acceptable pain at baseline predicts psychosocial deterioration at follow-up. Formal psychometric evaluation of the acceptable pain VAS item would clarify the construct being measured. Critical next steps include concurrent assessment of pain catastrophizing, pain self-efficacy, trait negative affectivity, and treatment expectations. Replication in diagnosis-specific populations and primary care settings would strengthen the evidence base.

## Conclusions

Self-reported acceptable pain intensity is associated with psychosocial symptom burden, specifically somatization, anxiety, sleep impairment, and perceived stress, but not depression or functional disability, in chronic pain patients, after adjustment for pain severity and trauma history. Higher acceptable pain is linked to elevated somatization, anxiety, sleep impairment, and perceived stress, although the incremental contribution is small. This study does not validate acceptable pain as a screening tool; rather, it indicates that higher self-reported acceptable pain should not be assumed to reflect adaptive acceptance, because it is consistently associated with psychosocial burden beyond pain severity. Further construct validation, including concurrent assessment against established pain-acceptance measures, is needed.

## Supplementary Information

Below is the link to the electronic supplementary material.

Supplementary Material 1

## Data Availability

The dataset analysed during the current study is publicly available in the Zenodo repository, https://doi.org/10.5281/zenodo.7498710.

## Abbreviations

VAS: visual analogue scale
PHQ-15: Patient Health Questionnaire-15 somatic symptom severity scale
GAD-7: Generalized Anxiety Disorder-7 scale
PHQ-Stress: Patient Health Questionnaire stress module
PHQ-9: Patient Health Questionnaire-9 depression scale
PDI: Pain Disability Index
CPAQ: Chronic Pain Acceptance Questionnaire
PASS: Patient Acceptable Symptom State
HKF-R 10: Heidelberg Pain Chronification Screening
NA: negative affectivity
PTSD: posttraumatic stress disorder
CI: confidence interval
