# Supplementary material for: Self-reported acceptable pain intensity is associated with psychosocial burden beyond pain severity in chronic pain patients: a cross-sectional study
Source: BMC Psychol. 2026 Oct 13;14:1473. doi: 10.1186/s40359-026-05731-8 (PMC13663201; doi:10.1186/s40359-026-05731-8)
Supplement: Supplementary file 1 [file 40359_2026_5731_MOESM1_ESM.docx]

**Supplementary Material**

*Self-Reported Acceptable Pain Intensity Is Associated With Psychosocial Burden Beyond Pain Severity in Chronic Pain Patients: a cross-sectional study*

**Table S1.** Benjamini–Hochberg false discovery rate correction for the six acceptable pain associations.

| **Outcome** | **Raw p** | **BH-adjusted p** | **Significant** |
| --- | --- | --- | --- |
| Somatization (PHQ-15) | <.001 | <.001 | Yes |
| Anxiety (GAD-7) | .002 | .003 | Yes |
| Sleep impairment (VAS) | .002 | .003 | Yes |
| Perceived stress (PHQ-Stress) | .002 | .003 | Yes |
| Depression (PHQ-9) | .054 | .065 | No |
| Disability (PDI) | .825 | .825 | No |

*Note.* Raw p-values are for the acceptable pain coefficient in each primary multivariable model (covariates: age, sex, mean, maximal, and current pain intensity, trauma category). BH-adjusted p-values control the false discovery rate across the six outcomes at q = 0.05. Significant = BH-adjusted p < .05.

**Table S2.** Sensitivity analyses for the acceptable pain coefficient (per 10 VAS) under alternative model specifications.

| **Outcome** | **Model A** mean pain only | **Model B** trauma dummy-coded | **Model C** extremes excluded |
| --- | --- | --- | --- |
| Somatization (PHQ-15) | 0.43 (<.001) | 0.36 (<.001) | 0.47 (<.001) |
| Anxiety (GAD-7) | 0.33 (<.001) | 0.28 (.003) | 0.27 (.024) |
| Sleep impairment (VAS) | 2.11 (<.001) | 1.64 (.003) | 2.40 (<.001) |
| Perceived stress (PHQ-Stress) | 0.26 (<.001) | 0.22 (.003) | 0.26 (.007) |
| Depression (PHQ-9) | 0.24 (.011) | 0.17 (.073) | 0.22 (.082) |
| Disability (PDI) | 0.20 (.433) | 0.06 (.828) | 0.47 (.138) |

*Note.* Each cell reports the unstandardized acceptable pain coefficient (per 10 VAS points) with its uncorrected p-value in parentheses. Model A replaces the three pain intensity covariates with mean pain only. Model B codes trauma category as three dummy variables (reference: no trauma) rather than ordinally. Model C excludes extreme acceptable pain values (acceptable pain = 0 and > 80; n = 745). All other covariates as in the primary models.

**Table S3.** Post-hoc analyses: trauma × acceptable pain interaction (Panel A) and trauma-stratified acceptable pain coefficients (Panel B).

| **Variable** | **B** | **95 % CI** | **p** | **ΔR²** |
| --- | --- | --- | --- | --- |
| *Panel A. Trauma category × acceptable pain interaction terms (n = 822)* | | | | |
| Somatization (PHQ-15) | +0.128 | [-0.03, 0.28] | .107 | 0.002 |
| Anxiety (GAD-7) | +0.165* | [0.01, 0.32] | .042 | 0.004 |
| Sleep impairment (VAS) | +0.511 | [-0.43, 1.45] | .288 | 0.001 |
| Perceived stress (PHQ-Stress) | +0.137* | [0.01, 0.26] | .032 | 0.005 |
| Depression (PHQ-9) | +0.335*** | [0.17, 0.50] | <.001 | 0.014 |
| Disability (PDI) | -0.120 | [-0.55, 0.31] | .586 | 0.000 |
| *Panel B. Acceptable pain coefficients (per 10 VAS) stratified by trauma group* | | | | |
| ***Depression (PHQ-9)*** | | | | |
| No trauma (n = 231) | -0.050 | [-0.42, 0.32] | .788 | — |
| Accidental (n = 210) | -0.032 | [-0.39, 0.33] | .861 | — |
| Interpersonal (n = 282) | +0.351* | [0.00, 0.70] | .049 | — |
| PTSD-level (n = 99) | +0.673** | [0.19, 1.15] | .006 | — |
| ***Somatization (PHQ-15)*** | | | | |
| No trauma (n = 231) | +0.400* | [0.08, 0.72] | .015 | — |
| Accidental (n = 210) | +0.249 | [-0.09, 0.59] | .151 | — |
| Interpersonal (n = 282) | +0.225 | [-0.12, 0.57] | .205 | — |
| PTSD-level (n = 99) | +0.715** | [0.21, 1.22] | .006 | — |

*Note.* Panel A reports the coefficient of the multiplicative interaction term (trauma category × acceptable pain per 10 VAS) added to each of the six primary multivariable models (covariates: age, sex, mean pain, maximal pain, current pain, trauma category, acceptable pain). Panel B reports the acceptable pain coefficient (per 10 VAS) within each trauma category, with the same covariates except trauma category. B = unstandardized regression coefficient; CI = confidence interval; ΔR² = incremental variance explained by the interaction term. Bonferroni correction across the six interaction tests in Panel A was applied at α = .008. Significance symbols denote uncorrected *p*-values. ****p* < .001; ***p* < .01; **p* < .05; †*p* < .10.

**Table S4.** Descriptive comparison of acceptable pain subgroups (n = 822).

| **Variable** | **Acc = 0** (n = 58) | **Acc 1–49** (n = 698) | **Acc ≥ 50** (n = 66) | **p¹** | **p²** |
| --- | --- | --- | --- | --- | --- |
| Age (years) | 46.1 (19.1) | 54.4 (15.7) | 53.1 (17.0) | .002** | .570 |
| Female sex, n (%) | 39 (67.2) | 479 (68.6) | 42 (63.6) | .831 | .425 |
| Mean pain (VAS) | 55.1 (29.3) | 57.8 (21.8) | 77.2 (20.1) | .487 | <.001*** |
| Maximal pain (VAS) | 71.3 (29.2) | 75.6 (20.8) | 85.6 (16.6) | .283 | <.001*** |
| Current pain (VAS) | 41.6 (31.7) | 51.1 (26.9) | 74.5 (25.2) | .030* | <.001*** |
| Acceptable pain (VAS) | 0.0 (0.0) | 23.6 (13.0) | 68.1 (18.5) | <.001*** | <.001*** |
| Somatization (PHQ-15) | 10.1 (5.3) | 12.4 (5.2) | 15.8 (6.0) | .002** | <.001*** |
| Anxiety (GAD-7) | 6.0 (4.9) | 7.8 (5.1) | 10.6 (5.2) | .007** | <.001*** |
| Sleep impairment (VAS) | 45.1 (36.1) | 51.6 (31.9) | 73.9 (29.6) | .189 | <.001*** |
| Perceived stress (PHQ-Stress) | 5.1 (3.8) | 6.7 (4.0) | 8.5 (4.3) | .002** | .001** |
| Depression (PHQ-9) | 9.6 (5.8) | 11.0 (5.5) | 14.6 (5.7) | .070† | <.001*** |
| Disability (PDI) | 31.7 (17.5) | 35.7 (14.9) | 42.6 (17.2) | .097† | .002** |
| *Trauma category, n (%)ᵃ* | | | | .005** | .030* |
| No trauma | 28 (48.3) | 187 (26.8) | 16 (24.2) |  |  |
| Accidental | 8 (13.8) | 183 (26.2) | 19 (28.8) |  |  |
| Interpersonal | 17 (29.3) | 249 (35.7) | 16 (24.2) |  |  |
| PTSD-level | 5 (8.6) | 79 (11.3) | 15 (22.7) |  |  |

*Note.* Values are mean (standard deviation) for continuous variables and n (%) for categorical variables. The Acc 1–49 group serves as the reference. p¹ = Welch’s t-test comparing Acc = 0 vs. Acc 1–49. p² = Welch’s t-test comparing Acc ≥ 50 vs. Acc 1–49. ᵃFor the categorical trauma variable, p-values are from χ² tests comparing the full four-category distribution between the respective group and the reference (all expected cell counts > 5). These group comparisons are unadjusted; however, the elevated psychosocial scores in the Acc ≥ 50 group relative to the reference remained significant after adjustment for all three pain intensity measures for somatization, anxiety, sleep impairment, perceived stress, and depression (all adjusted p < .05), but not for disability (adjusted p = .95), indicating that the subgroup differences are not attributable to higher pain intensity alone. Acc = self-reported acceptable pain intensity (VAS, 0–100). VAS = visual analogue scale. PHQ-15 = Patient Health Questionnaire somatic symptom severity scale; GAD-7 = Generalized Anxiety Disorder scale; PHQ-Stress = Patient Health Questionnaire stress module; PHQ-9 = Patient Health Questionnaire depression scale; PDI = Pain Disability Index. Significance symbols denote uncorrected *p*-values. ****p* < .001; ***p* < .01; **p* < .05; †*p* < .10.
